# Effectiveness and cost-effectiveness of telephone consultations for fever or gastroenteritis using a formalised procedure in general practice: study protocol of a cluster randomised controlled trial

**DOI:** 10.1186/s13063-016-1585-9

**Published:** 2016-09-22

**Authors:** Paul-Georges Reuter, Thibaut Desmettre, Sabine Guinemer, Olivier Ducros, Stéphane Begey, Agnès Ricard-Hibon, Laurianne Billier, Océane Grignon, Isabelle Megy-Michoux, Jean-Noël Latouff, Adeline Sourbes, Julien Latier, Isabelle Durand-Zaleski, Frédéric Lapostolle, Eric Vicaut, Frédéric Adnet

**Affiliations:** 1 Service des Urgences et Service d’Aide Médicale Urgente, Centre Hospitalier Universitaire Avicenne, Assistance Publique-Hôpitaux de Paris, 125 rue de Stalingrad, 93009 Bobigny Cedex, France; 2 Université Paris 13, Sorbonne Paris Cité, EA 3509 Bobigny, France; 3 Urgences et SAMU 25 – Centre Hospitalier Régional Universitaire de Besançon, 1 Bd Fleming, 25030 Besançon Cedex, France; 4 Université de Franche Comté-Bourgogne, UMR 6249 CNRS/UFC, 1 Bd Fleming, 25030 Besançon Cedex, France; 5 SAMU-SMUR 95, Centre Hospitalier René Dubos, 6 Avenue de l’Île de France, 95300 Pontoise, France; 6 Association COmtoise de REgulation Libérale, CHRU de Besançon, 1 Bd Fleming, 25030 Besançon Cedex, France; 7 SAMU 44, PHU Urgences – Médecines – Soins Critiques, Centre Hospitalo-Universitaire de Nantes, 1 quai Moncousu, 44 093 Nantes Cedex, France; 8 SAMU SMUR Urgences, Centre Hospitalier Châteauroux, 216 avenue de Verdun, 36000 Châteauroux, France; 9 SAMU 82, Centre Hospitalier de Montauban, 100 rue Léon Vladel, 82000 Montauban, France; 10 Assistance Publique-Hôpitaux de Paris, URC Eco, Paris, France; 11 Inserm, ECEVE, U1123 Paris, France; 12 Unité de Recherche Clinique, Saint Louis – Lariboisière – Fernand Widal University Hospital, AP-HP, Paris, France

**Keywords:** Telephone consultation, Primary care, Cost-effectiveness, Satisfaction, Compliance, Fever, Gastroenteritis, Cluster randomised controlled trial

## Abstract

**Background:**

Telephone consultations in general practice are on the increase. However, data on their efficiency in terms of out-of-hours general practitioner (GP) workload, visits to hospital emergency departments (ED), cost, patient safety and satisfaction are relatively scant. The aim of this trial is to assess the effectiveness of telephone consultations provided by French emergency call centres in patients presenting with isolated fever or symptoms of gastroenteritis, mainly encountered diseases.

**Methods/design:**

This is a prospective, open-label, multicentre, pragmatic, cluster randomised clinical trial of an estimated 2880 patients making an out-of-hours call to one of six French emergency call centres for assistance with either fever or symptoms of gastroenteritis without seriousness criteria. Each call is handled by a call centre physician. Out-of-hours is 8 p.m. to 7.59 a.m. on weekdays, 1 p.m. to 7.59 a.m. on Saturdays and round-the-clock on Sundays and school holidays. Patients will be enrolled over 1 year.

In the intervention arm, a telephone consultation based on a protocol, the formal Telephone Medical Advice (fTMA), is offered to each patient calling. This protocol aims to overcome a physical consultation during out-of-hours periods. It offers reassurance and explanations, advice on therapeutic management which may include, in addition to hygiene and diet measures, a telephone prescription of antipyretic, analgesic, rehydration medication or others, and recommendations on surveillance of the patient and any action to be taken. The patient is invited to call again if the condition worsens or new symptoms develop and to make an appointment with their family GP during office hours. In the control arm, the call centre physician handles calls as usual. This physician can carry out a telephone consultation with or without a telephone prescription, dispatch an on-duty GP, the fire brigade or an ambulance to the patient, or refer the patient to an on-duty physician or to the ED. Each patient will receive a follow-up call on day 15.

The primary endpoint is the frequency of out-of-hours, face-to-face GP consultations or visits to the ED during the 15 days following the index call. The secondary endpoints measured on day 15 are the number of stays in intensive care, the number of hospital admissions, the number of interventions by the fire brigade, emergency medical and ambulance services, the number and length of prescribed sick-leave episodes, all-cause mortality, morbidity, clinical outcome, patient compliance, patient satisfaction, the number of renewed calls to the call centre, the number of patients receiving multiple face-to-face GP consultations and costs incurred.

**Discussion:**

This trial will assess the effectiveness and the cost-effectiveness of a formalised response to calls for assistance with fever or symptoms of gastroenteritis without seriousness criteria.

**Trial registration:**

ClinicalTrials.gov Identifier: NCT02286245, registered on 9 September 2014.

## Background

Medical advice provided over the telephone is increasingly common in general practice [1–3]. Most calls made to medical call centres concern issues relating to general practice, in particular out-of-hours care. A widespread response to such calls is a telephone consultation [1]. The advice given may relate to action to be taken in the home including surveillance, and may involve telephone prescription and may invite the caller to consult a general practitioner (GP) [4].

Few prospective studies have addressed the effectiveness of advice given over the telephone in primary care in terms of patient compliance, satisfaction and benefit. Moreover, the impact of telephone advice on health costs has not been properly evaluated. A Cochrane review, which identified only five comparative randomised studies on the possibly superior efficacy of telephone consultations over usual care, concluded that such advice had little impact in terms of fewer visits to the family physician or to the hospital emergency department (ED) [1]. The review recommended that high-evidence level studies be conducted with patient satisfaction, safety and cost as endpoints.

In a study in the UK, in which out-of-hours calls to a general practice cooperative were randomised into two groups – namely, telephone consultations with a nurse versus usual general practice care – there was a reduction in costs arising from reduced emergency admissions to hospital in the nurse consultation group [5]. In a study in two urban practices in Scotland, supported by a UK study, telephone consultations for same-day appointments saved general practitioner (GP) time but led to a higher rate of reconsultations in the 2 weeks that followed [6, 7]. On the other hand, the introduction of telephone triage in another UK study significantly reduced by 39 % the demand for face-to-face consultations for patients seeking same-day appointments [8]. The number of ED visits does not seem to be related to telephone consultations [6–9]. The impact on cost seems negligible [7]. In a recent randomised controlled trial conducted in the UK, telephone triage by a nurse or a GP increased the primary care contact compared with usual care for patients calling for a same-day appointment [10, 11]. However, this trial did not focus on out-of-hours periods.

In France, calls for medical emergency are managed by the Service d’Aide Médicale Urgente (SAMU). Calls come from patients with severe symptoms, such as chest main, major trauma, and stroke, and from primary care [12]. In this last subgroup, the most frequently encountered requests for assistance in SAMUs relate to fever and symptoms of gastroenteritis [13, 14]. In response to a call, the medical dispatcher can give medical advice including recommending going into a medical care facility or can send a first aid team (usually the fire brigade), a light ambulance (drivers with first aid training), a GP trained in emergency care or a mobile intensive care unit with an emergency physician. Our study focussed on the management of calls for primary care.

The hypothesis of our study is that the medical advice concluding telephone consultations will provide a benefit to the individual and to society. We postulate that telephone consultations based on a protocol, the formal Telephone Medical Advice (fTMA), might offer an appropriate and effective response to demands made to a call centre. The aim of our study was to determine whether a fTMA can provide a reliable response, in terms of effectiveness and efficiency, in patients presenting with isolated fever or symptoms of gastroenteritis during the out-of-hours period.

## Methods/design

### Design

**Fig. 1:**
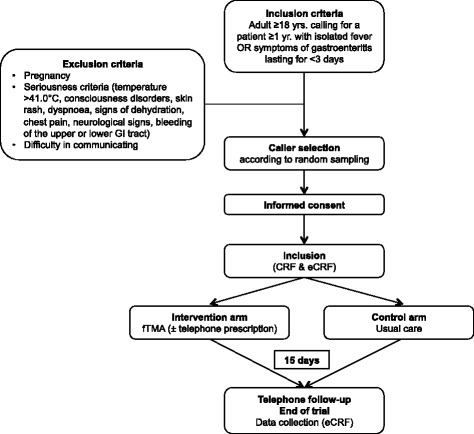
Flowchart

This is a prospective open-label, multicentre, pragmatic, cluster randomised clinical trial involving six participating centres (SAMU). The centres are randomised into two arms (1:1) according to call management method, either an offer of fTMA (intervention arm) or usual after-hours medical care (control arm). Out-of-hours is 8 p.m. to 7.59 a.m. on weekdays, 1 p.m. to 7.59 a.m. on Saturdays and round-the-clock on Sundays and school holidays. Callers/patients will be randomly selected before inclusion (see Fig. 1 for the study flowchart).

### Participants

Each call made after hours for either fever (i.e. a body temperature above 38.0 °C) or symptoms of gastroenteritis (nausea and/or vomiting and/or diarrhoea) will be included. The caller has to be over 18 years of age or to be calling regarding a patient aged over 1 year if the caller is not the patient. Fever and gastroenteritic symptoms must have occurred within the previous 72 h.

Exclusion criteria are (1) pregnancy if the caller is the patient, (2) seriousness criteria (i.e. temperature above 41.0 °C, consciousness disorders, skin rash, dyspnoea, signs of dehydration, chest pain, neurological signs, bleeding of the upper or lower gastrointestinal tract and (3) difficulty in communicating (uncommunicative patient, language barrier).

The caller will be informed of the study at the time of their call. Their informed consent will be requested. The caller will receive a copy of the information sheet by mail.

### Intervention

Control arm call centres will handle calls as usual. The call centre physician can carry out a telephone consultation with or without a telephone prescription, dispatch an on-duty GP, the fire brigade or an ambulance to physically examine the patient, or refer the patient to an on-duty physician or to the hospital ED.

**Fig. 2:**
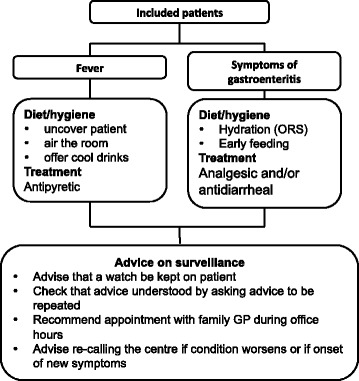
Trial protocol

The fTMA arm call centres will always offer medical advice over the telephone according to the following protocol (Fig. 2):

1. Reassurance and explanations: addressing nonserious symptoms, the call centre physician provides reassurance to the caller/patient and informs them that home management is possible
2. Therapeutic management (this may include telephone prescribing [4]): (a) in cases of fever, three hygiene and dietary measures are recommended: removal of the patient’s clothing, airing the room, and offering frequent cool drinks. An antipyretic agent may be prescribed over the telephone if the fever is poorly tolerated. Only one of two drugs should be prescribed, either paracetamol or ibuprofen, the dose depending on the patient’s bodyweight, (b) in cases of symptoms of gastroenteritis, the main measure is early rehydration, i.e. taking small amounts of water at frequent and regular intervals. The call centre physician should explain that this reduces vomiting. An oral rehydration solution (one sachet in 200 mL of water regardless of brand) is recommended in children. The patient should be advised to eat 4 to 6 hours after rehydration. Recommended foods are carrots, rice, apples, bananas, quince, potatoes, and lean meat. Fibres, citrus fruits and cooked fats are to be temporarily avoided. Racecadotril (Tiorfan®), three times a day, can be teleprescribed to decrease bowel movements, the dosage depending on the patient’s body weight. In cases of fever or pain, paracetamol can be prescribed as a function of bodyweight
3. Advice on surveillance and any action to be taken: the purpose of surveillance is to ensure good tolerance of symptoms. In cases of fever, this is not a return to a normal body temperature. The caller must repeat the advice and medical prescription to the call centre physician to show that they have understood. The patient must make an appointment with their GP during office opening hours. The patient must always call again if the fever persists despite treatment, if their condition worsens, or if further symptoms appear

Patients for whom the call centre physician has to call someone out or refer the patient to a GP on duty or to the ED will be identified and analysed.

### Outcomes

The primary endpoint is the percentage of patients receiving an out-of-hours, face-to-face consultation with a GP, or who are admitted to hospital, during the 2 weeks following their call to the centre. The motive for the consultation or hospital admission should be the same as the motive initially prompting the call. Secondary endpoints are the following variables measured over the 2 weeks: number of stays in intensive care, number of hospital admissions, number of interventions by the fire brigade, emergency medical and ambulance services, number and length of prescribed sick leave episodes, all-cause mortality, morbidity, clinical outcome, patient compliance, patient satisfaction, number of renewed calls to the call centre, number of patients receiving multiple face-to-face consultations and costs incurred.

### Economic evaluation

Only health care (acute) resources are considered. Intervention costs are obtained with a bottom-up microcosting approach that identifies all relevant cost components of the telephone intervention and values each of those components for all individual patients using the following variables: duration, staff, and equipment. Consultations, sick leave, drugs and other resources and emergency interventions for each patient are recorded in the electronic Case Report Form (eCRF) or retrieved from the hospital databases. The prices of drugs, consultations and an emergency ambulance are based on national tariffs. Hospitalisation costs are estimated from the average national cost of each patient’s diagnosis-related groups weighted with their actual length of stay and resources used during their hospitalisation (intensive care, blood transfusion, etc.). The time horizon is 15 days.

For the purpose of the cost-effectiveness analysis, we define a composite endpoint of adverse events combining hospital admissions, emergency visits and 15-day mortality. A cost-effectiveness analysis is conducted to assess incremental cost per adverse event averted. The cost-effectiveness analysis focusses on estimation of the joint density of cost and effect differences and quantification of uncertainty surrounding the incremental cost-effectiveness ratio. The absence of a significant difference in either cost or effectiveness, or both, does not preclude the presentation of such data on the cost-effectiveness plane [15, 16].

### Data collection

Inclusion becomes effective when the person telephoning the call centre agrees to data collection and to a follow-up call.

The Case Report Form (CRF) is completed by the investigator at the call centre. It gives the caller’s and/or patient’s identification details (initials, date of birth, sex, relationship to the patient and the centre’s allocated patient number), inclusion and exclusion criteria, the medical indication for the call (fever or symptoms of gastroenteritis), and the caller’s reason for calling (request for advice, for a physician, etc.). The CRF also contains data on the following: how the call is handled by the centre (time taken, number of calls on hold whilst handling the call, the patient’s history, handling according to study arm), self-evaluation of performance by the call centre’s physician, foreseeable delay before any intervention by an on-duty GP at the caller’s home, and the names and postal codes of localities with the closest emergency facilities, duty GP and duty pharmacist.

A follow-up telephone call will be made 15 ± 4 days after the index telephone call (D0), regardless of study arm, by a clinical trial technician (CTT) trained by the trial investigator at the coordinating centre. The CTT will record the following: date, time and duration of follow-up call, the patient’s environment (rural or urban), answers to a satisfaction questionnaire on call handling on D0, a clinical evaluation of the patient (compliance, course), and all data required for the health economic evaluation. If the patient is underage and/or unable to answer the questions, only the caller will be interviewed. The patient will be considered lost-to-follow-up if there is no reply after a series of 15 calls.

All data will be archived as an eCRF under the responsibility of the Clinical Research Unit of Lariboisière-Saint Louis, Paris, France. The paper versions of the CRF will be kept in safe keeping in each centre for a period of 15 years.

### Patient information

The patient will be included after the call centre’s physician has obtained the caller’s informed consent. The patient will be sent an information sheet by mail.

### Pilot study

A pilot study of 570 calls conducted from January to June 2011 at the Seine-Saint-Denis call centre demonstrated the feasibility of caller recruitment and of patient follow-up by telephone. The percentage of refusals-to-participate and lost-to-follow-up was 15 %.

### Sample size

We postulate that fTMA will reduce the percentage of out-of-hours on-duty GP consultations or the number of visits to an ED during the 2 weeks following the index call. In the study by Lattimer et al., the percentage of hospital admissions and ED visits was 12 % after a nurse telephone consultation [17]. In a parallel study, Thompson et al. reported a 21 % versus a 33 % overnight call rate resulting in a home visit by a duty GP in the nurse telephone intervention arm versus the control arm [18]. We estimate that the percentage of out-of-hours consultations and ED visits (our primary endpoint) will be 50 % in the control group. We aim for an absolute reduction of at least 10 % (i.e. from 50 to 40 %) in the intervention arm for each indication (fever/gastroenteritis).

On the assumption of a relatively low weak design effect due to cluster randomisation (about 1.5), and considering a loss of follow-up of 15 %, it is estimated that a sample size of 663 patients/arm/indication will be required for 85 % power and a 5 % alpha risk. If account is taken of an attrition rate of about 8 %, 720 patients will be required (1440/indication, i.e. a total of 2880 patients). This sample size should provide enough power and precision for subgroup analyses (season versus diurnal cycle, weekend versus weekdays).

Inclusions will take place over a full year in order to obtain a representative sample. Each centre will thus include about 240 patients for each indication. A random sample of four patients per week will be drawn outside of an epidemic (about 42 weeks) and of eight patients per week during an epidemic (about 10 weeks) in each centre for each indication. For feasibility reasons, the first patient whose call is closest to the dates and times that will have been randomly drawn for each week of the year of study will be included for each indication.

### Statistical analyses

Analyses will be intent-to-treat (ITT). Any missing values for the primary endpoint will be computed by the multiple imputation method. In which case, the robustness of the conclusions made on observed cases will be verified and a possible discrepancy will be analysed based on any biases in data collection that may have been identified.

Descriptive analyses will provide the following information for each continuous variable: mean value, standard deviation, 95 % confidence intervals (95 % CI), minimum, 1st quartile, median, 3rd quartile and maximum, and number of missing observations. Categorical variables will be summarised in frequency tables (with 95 % CI). Intracluster correlation coefficients will be calculated.

The primary endpoint will be tested using the Generalised Estimating Equations model, account being taken of the cluster trial design. Secondary continuous endpoints will be analysed with a mixed-model ANOVA including a random centre effect. For categorical variables, treatment effect will be tested using the Generalised Estimating Equations model, account being taken of the cluster trial design. All tests will be two-tailed with a 5 % alpha risk. All statistical tests will be performed using SAS version 9.2 software.

The health economic analysis will consider total cost, hospital costs, ambulatory costs, cost borne by the family and costs to society. Analysis of management costs in both study arms will be ITT. Cost comparisons will be performed using appropriate statistical methods after determining the type of distribution (normal, log normal or beta).

## Discussion

This trial will assess the effectiveness and the cost-effectiveness of a formalised response to calls made for assistance with fever or symptoms of gastroenteritis without seriousness criteria. The inclusion period ended in July 2016. The follow-up is still running and the economic evaluation will start shortly. Two of the centres had several difficulties in enrolling patients. One centre because of a lack of calls made for symptoms of gastroenteritis and the other because of an organisational problem not attributable to the trial. To analyse a potential seasonality effect, the recruitment period stopped at 12 months, as described in the methods section, even if centres had not reach the number of included patients.

### Final report

The final report will adhere to the Consolidated Standards of Reporting Trials (CONSORT) extension for cluster trials.

## Trial status

The inclusion period ended in July 2016.

## Abbreviations

eCRF: Electronic case report form
ED: Emergency Department
fTMA: Formal telephone medical advice
GI: Gastrointestinal
GP: General practitioner
ITT: Intent-to-treat
